# Early Detection of Ecosystem Regime Shifts: A Multiple Method Evaluation for Management Application

**DOI:** 10.1371/journal.pone.0038410

**Published:** 2012-07-10

**Authors:** Martin Lindegren, Vasilis Dakos, Joachim P. Gröger, Anna Gårdmark, Georgs Kornilovs, Saskia A. Otto, Christian Möllmann

**Affiliations:** 1 Scripps Institution of Oceanography, University of California San Diego, La Jolla, California, United States of America; 2 National Institute of Aquatic Resources, Technical University of Denmark, Charlottenlund, Denmark; 3 Department of Aquatic Ecology & Water Quality Management, Wageningen University, Wageningen, The Netherlands; 4 Institute for Sea Fisheries, von-Thünen Institute, Federal Research Institute for Rural Areas, Forestry and Fisheries, Hamburg, Germany; 5 Institute for Bio-Sciences, University of Rostock, Rostock, Germany; 6 Department of Aquatic Resources, Swedish University of Agricultural Sciences, Institute of Coastal Research, Öregrund, Sweden; 7 Department of Fish Resources Research, Institute of Food Safety, Animal Health and Environment, Riga, Latvia; 8 Institute for Hydrobiology and Fisheries Science, University of Hamburg, Hamburg, Germany; National Institute of Water & Atmospheric Research, New Zealand

## Abstract

Critical transitions between alternative stable states have been shown to occur across an array of complex systems. While our ability to identify abrupt regime shifts in natural ecosystems has improved, detection of potential early-warning signals previous to such shifts is still very limited. Using real monitoring data of a key ecosystem component, we here apply multiple early-warning indicators in order to assess their ability to forewarn a major ecosystem regime shift in the Central Baltic Sea. We show that some indicators and methods can result in clear early-warning signals, while other methods may have limited utility in ecosystem-based management as they show no or weak potential for early-warning. We therefore propose a multiple method approach for early detection of ecosystem regime shifts in monitoring data that may be useful in informing timely management actions in the face of ecosystem change.

## Introduction

Transitions between alternative states, i.e., regime shifts, have been shown to occur across an array of complex systems [1], [2], including ecosystems [3]. Our ability to identify abrupt shifts in real ecosystems has improved through advances in theory and statistical methods [4]. However, these methods are primarily designed to detect regime shifts once having occurred. Recent theoretical studies suggest that several indicators may be used as early-warnings of an approaching transition [3]. Although needed for short-term management efforts to maintain key ecosystem goods and services, empirical applications of early detection of abrupt shifts in real ecosystems have so far mainly been limited to experimental studies [5], [6] or paleo-climatic reconstructions over vast temporal scales [7], [8].

Several early-warning indicators have been proposed to describe the temporal dynamics of complex systems close to a critical transition [3]. The basic rationale behind these indicators lies in the fact that the recovery of a system to equilibrium after a perturbation becomes slower close to a transition [9]. This phenomenon is known as ‘critical slowing down’ [10] and causes the variance and autocorrelation in the fluctuations of a system to increase prior to a regime shift [3], [11], [12]. In addition, the spatial dynamics of complex systems may also change close to a transition, where alterations in the spatial patterns of variance and correlation of key ecological features may serve as a complimentary set of early-warning indicators [13]–[15]. Although the merit of these indicators is that they can be detected across an array of ecosystems and types of transitions [16], their disadvantage is that they require long time series of high resolution for their estimation. Moreover, the potential for early-detection in practice is based on the assumption that the time series accurately represent the response of the ecosystem around its present equilibrium state [3]. Since ecological monitoring records are typically of limited length, lack detailed information on spatial distribution patterns of key organisms, and often include substantial measurement error, the practical use of any of the proposed early-warning indicators for ecosystem management may prove problematic. Given that these limitations can lead to both false positive and false negative signals [3], the use of multiple spatial and temporal indicators should ideally be considered [16] and alternative methods should be tested [12]. Yet, studies of early-warning signals in real ecosystems have so-far been restricted to only a narrow range of possible temporal [8] or spatial indicators [17].

**Figure 1:**
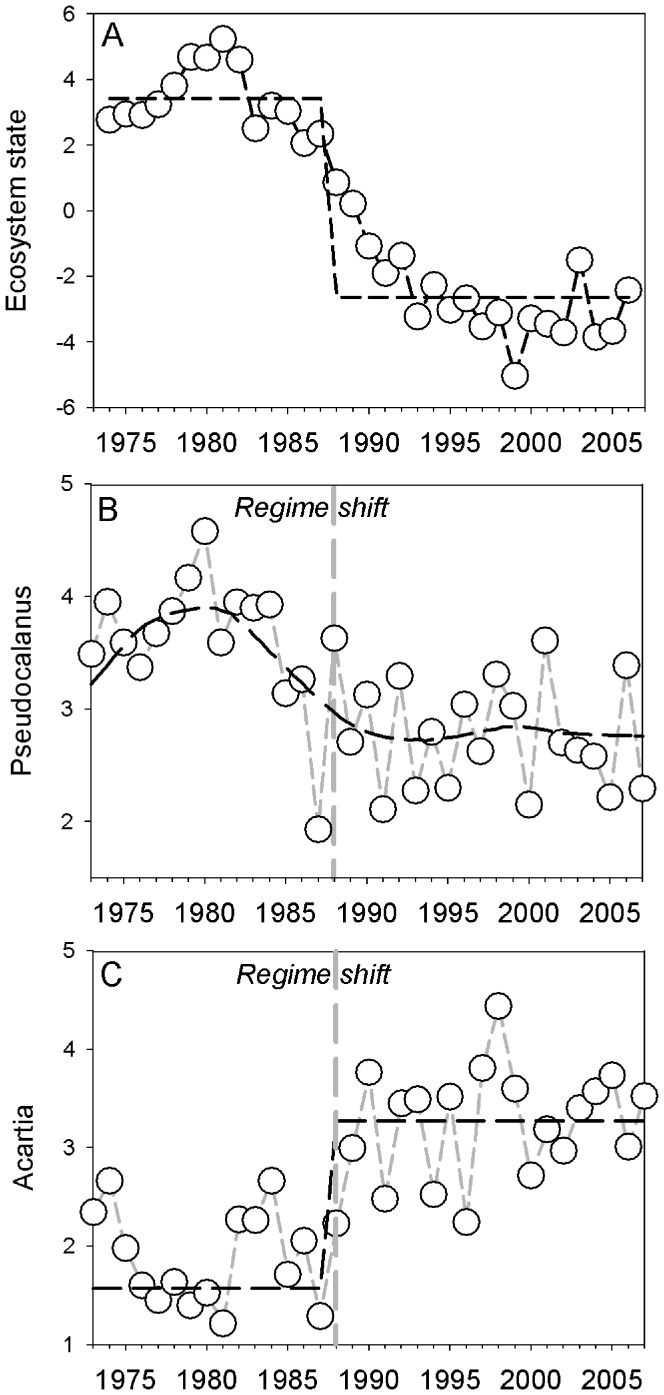
Ecosystem dynamics of the Central Baltic Sea. (A) The first principle component of a principle component analysis of biotic time series [20]. A significant break point based on the Sequential Regime Shift Detection Method illustrates the ecosystem regime shift in the late-1980s (dashed). Long-term dynamics of the selected early-warning indicators, *Pseudocalanus acuspes* (B) and *Acartia* spp. (C) during the corresponding time period (1974–2008) with the associated regime shift in 1988–1989 (grey). The black dashed lines illustrate two different types of transitions, i.e., gradual changes or a sudden (pulse) transitions, respectively.

Large-scale patterns of ecosystem change have been observed in marine ecosystems across the Northern hemisphere [18], [19], including the Baltic Sea [20]. A key question for marine management is whether these regime shifts could have been detected by early-warning indicators. Using real monitoring data of the copepods *Pseudocalanus acuspes* and *Acartia* spp., two key indicator species significantly contributing to the reorganization of the Baltic Sea ecosystem (Figure 1) [20], we here apply a set of methods for detecting trends and structural breaks in time series, i.e., (i) temporal and spatial indicators of critical slowing down, (ii) trend analysis and (iii) shiftograms, as alternative tools for early-detection of regime shifts. Lastly, we assess all early-detection methods, covering both temporal and spatial processes, in order to evaluate their practical use in forewarning the major regime shift that occurred in the Baltic Sea during the late 1980s (Figure 1) [20].

## Materials and Methods

### Ecosystem Characteristics and Data Considerations

The Baltic Sea is a large semi-enclosed sea (Figure S1), which due to its brackish nature is characterized by low species diversity, but high productivity. Climatic conditions since the late 1980s have significantly changed the living conditions for plant and animal populations inhabiting the area, caused by increasing temperatures and decreasing salinity and oxygen levels [21]. In addition to climate forcing, anthropogenic impact from overfishing and eutrophication likely contributed to the abrupt regime shift, which included trophic cascading involving several trophic levels [20], [22]. The regime shift occurred during a transition period between 1988 and 1993, where all external drivers were on extreme levels [20]. Given the difficulty of detecting the exact timing of regime shifts [4], we assume (for the purpose of this study) the major changes to have happened already in 1988 (Figure 1A).

**Figure 2:**
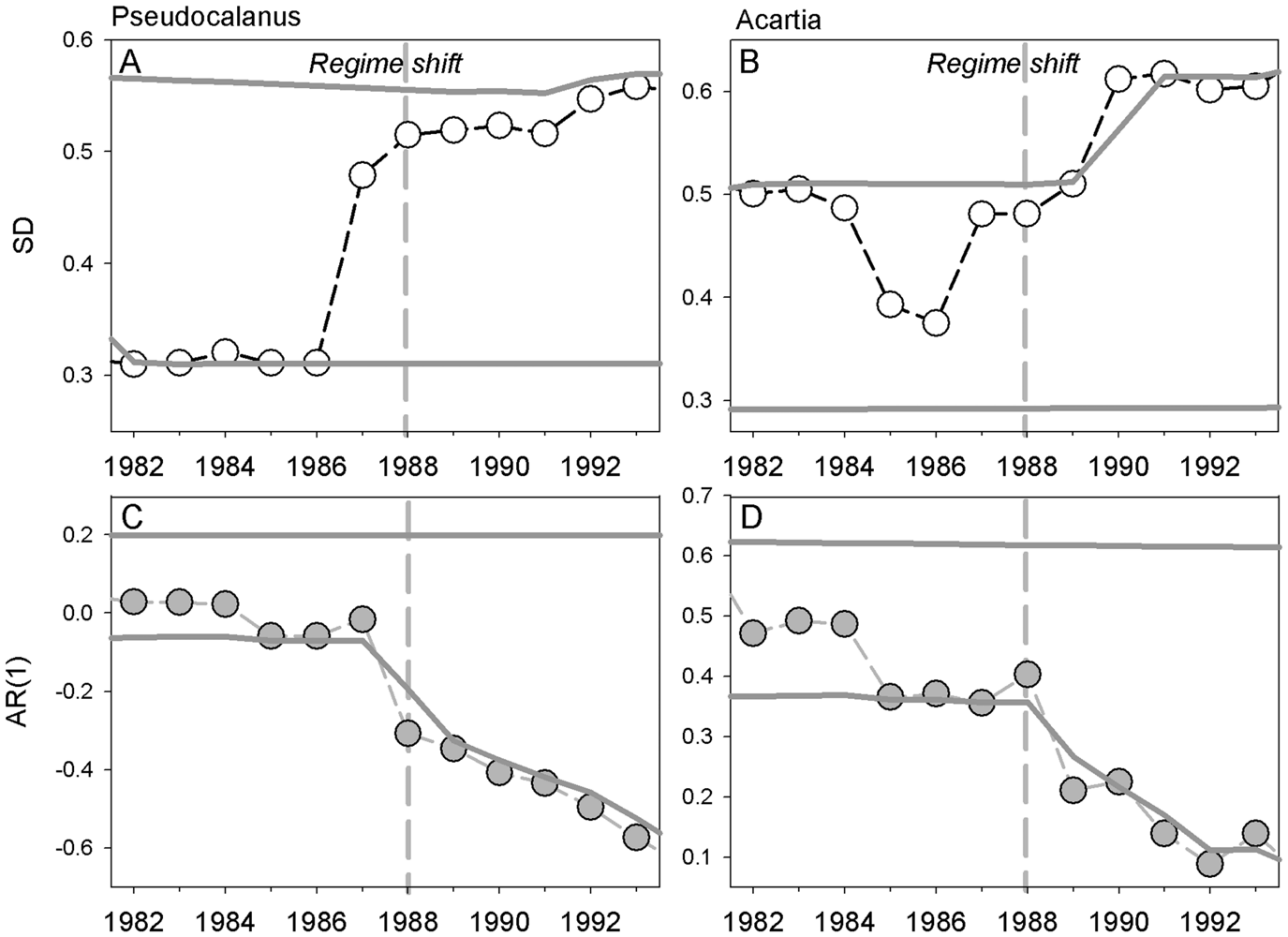
Temporal indicators of critical slowing down. Temporal variance (estimated as standard deviations, SD) and the first-order autocorrelation coefficient (AR(1)) for *Pseudocalanus acuspes* (A, C) and *Acartia* spp. (B, D) estimated within sliding windows of 10 years. Vertical dashed lines mark the timing of the regime shift in the late 1980s and grey solid lines the upper and lower 95% confidence intervals.

We used *Pseudocalanus acuspes* and *Acartia* spp., two key zooplankton species in the Central Baltic Sea food-web significantly contributing to the reorganization of the ecosystem, as indicators for the regime shift [20]. The population sizes and of these zooplankton species changed drastically during the regime shift (Figure 1B, C), which had strong implications for their major predators, such as larval cod [21], [23], [24], as well as the main planktivores in the ecosystem, i.e., herring (*Clupea harengus*) and sprat (*Sprattus sprattus*) [25]. Due to their pivotal role as mediators between lower trophic levels and the fish community [26], their rapid response to climate variability (high sensitivity to salinity and temperature, respectively), *Pseudocalanus acuspes* and *Acartia* spp. serve as suitable indicators for the ecosystem regime shift in the Central Baltic Sea. Furthermore, long-term temporally and spatially resolved monitoring programs [27], i.e., monthly coverage of sampling stations from 1960 and onwards, are available for both species, In order to assess the ability to detect abrupt regime shifts sufficiently in advance for management, we applied a set of early-detection methods on spatially aggregated and disaggregated (by sampling stations and areas; Fig. S1) data set of *Pseudocalanus acuspes* and *Acartia* spp. biomass covering the period 1960–2008. Since the primary aim of the study is to investigate and evaluate potential early-warning signals, we chose to focus on and present results on method performance during the time-period immediately preceding and following the regime shift (i.e., 1982–1993). We used data representing spring as this is the main reproductive season of the copepods [28], [29].

**Figure 3:**
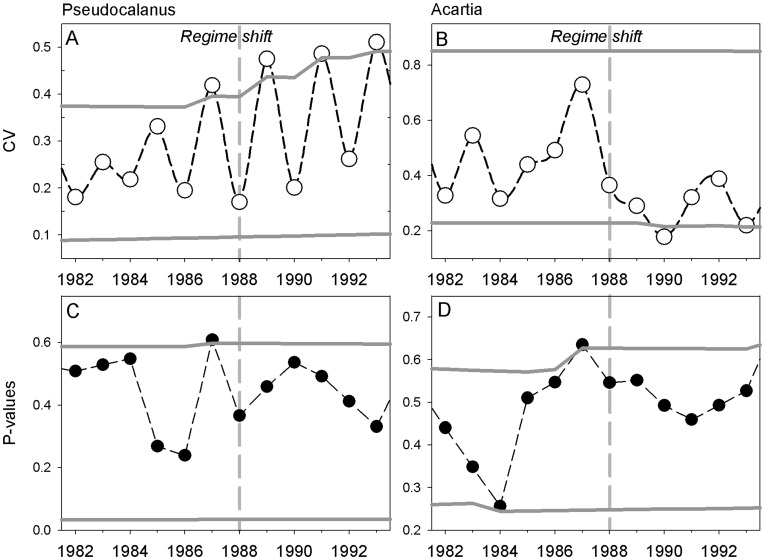
Spatial indicators of critical slowing down. The coefficient of variation (CV) and degree of spatial correlation for *Pseudocalanus acuspes* (A, C) and *Acartia* spp. (B, D) estimated as the mean significance (p-value) of spatial correlation across 8 randomly assigned stations. Vertical dashed lines mark the timing of the regime shift in the late 1980s and grey solid lines the upper and lower 95% confidence intervals.

### Indicators of Critical Slowing Down

We measured temporal variance (as the standard deviation; SD) and autocorrelation at lag-1 (AR(1)) in detrended time series of average spring biomass of *Pseudocalanus acuspes* and *Acartia* spp. within a 10, 15 and 20-year sliding window, following the approach by Dakos *et al*. [8]. Temporal variance [30] and autocorrelation at lag-1 [11] are expected to rise prior to a critical transition, as approaching an unstable equilibrium would theoretically lead to a larger degree of instability [8], [31]. While temporal variance was measured as the SD of detrended time-series (of equal sample size), a relationship between the mean of a population and its variance often exists when comparing different samples in space [17]. Hence, spatial variance was estimated by the coefficient of variation (CV; 100*SD/mean) [17] correcting for the mean across all stations from the entire sampling area (Figure S1). Similar to temporal variance it has been shown that spatial variance or the coefficient of variation increases before a catastrophic shift [13], [15]. In addition, spatial correlation may also change close to a shift [16]. Here, we estimated spatial correlation across sampling stations using the Moran’s I test [32]. In order to reduce bias from uneven sampling between years, we randomly selected 6, 8 and 10 stations per year and estimated the mean correlation coefficient and associated p-value for each year after 1000 random draws.

**Figure 4:**
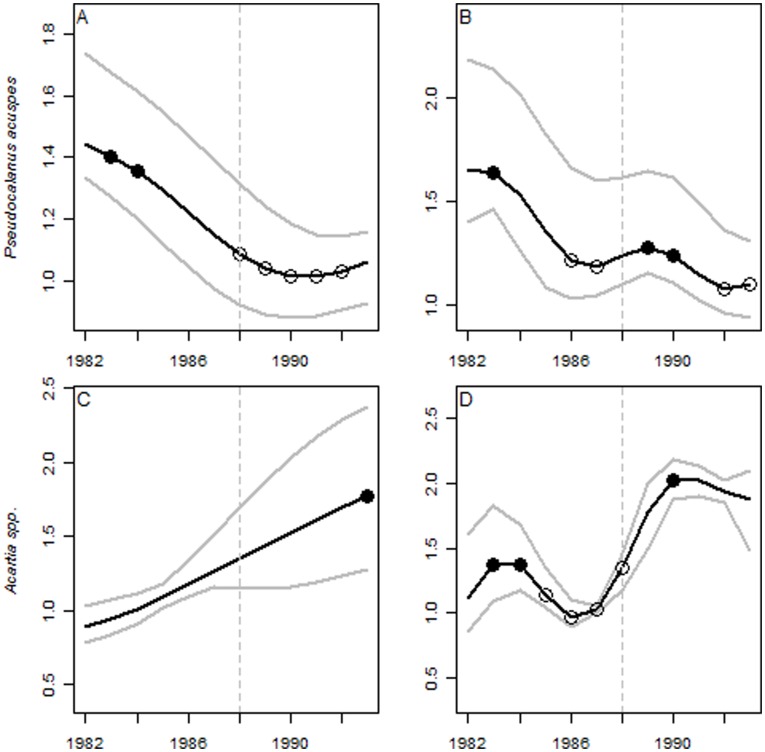
Trend analysis of indicator time series. Smoothed time series of *Pseudocalanus acuspes* and *Acartia* spp. based on GAMs using df = 10 (A, C) and df = 20 (B, D). Bootstrapped confidence intervals are shown by grey lines. Acceleration in the rate of change (slope) in each year are shown by statistically significant second derivatives (*f* ’’), where black and white dots represent major downward- and upward trends, respectively. Vertical dashed lines mark the timing of the regime shift in the late 1980s.

### Trend Analysis

In a second set of methods we applied statistical methods for assessing recent trends in the zooplankton time series. Although not specifically designed for early detection of regime shifts, the idea of using trend analysis as an early-warning signal lies in the possibility of detecting a slight increase in the rate of change (either in an upward or downward trend) in advance of a critical transition in an ecological time series. The approach is based on fitting non-linear Generalized Additive Models (GAM) [33] and estimating second derivatives (*f′′*) as a proxy for statistically significant acceleration in the rate of change (slope) of ecological time series [34], [35]. While the first approach relies on an *a priori* specified degree of smoothing [34], the second method [35] applies a routine for selecting optimal numbers of regression splines (degree of smoothing; df). In order to reduce potential bias due to the selection of regression splines, we performed the trend analysis using two levels of degrees of freedom (df = 10 and 20).

**Figure 5:**
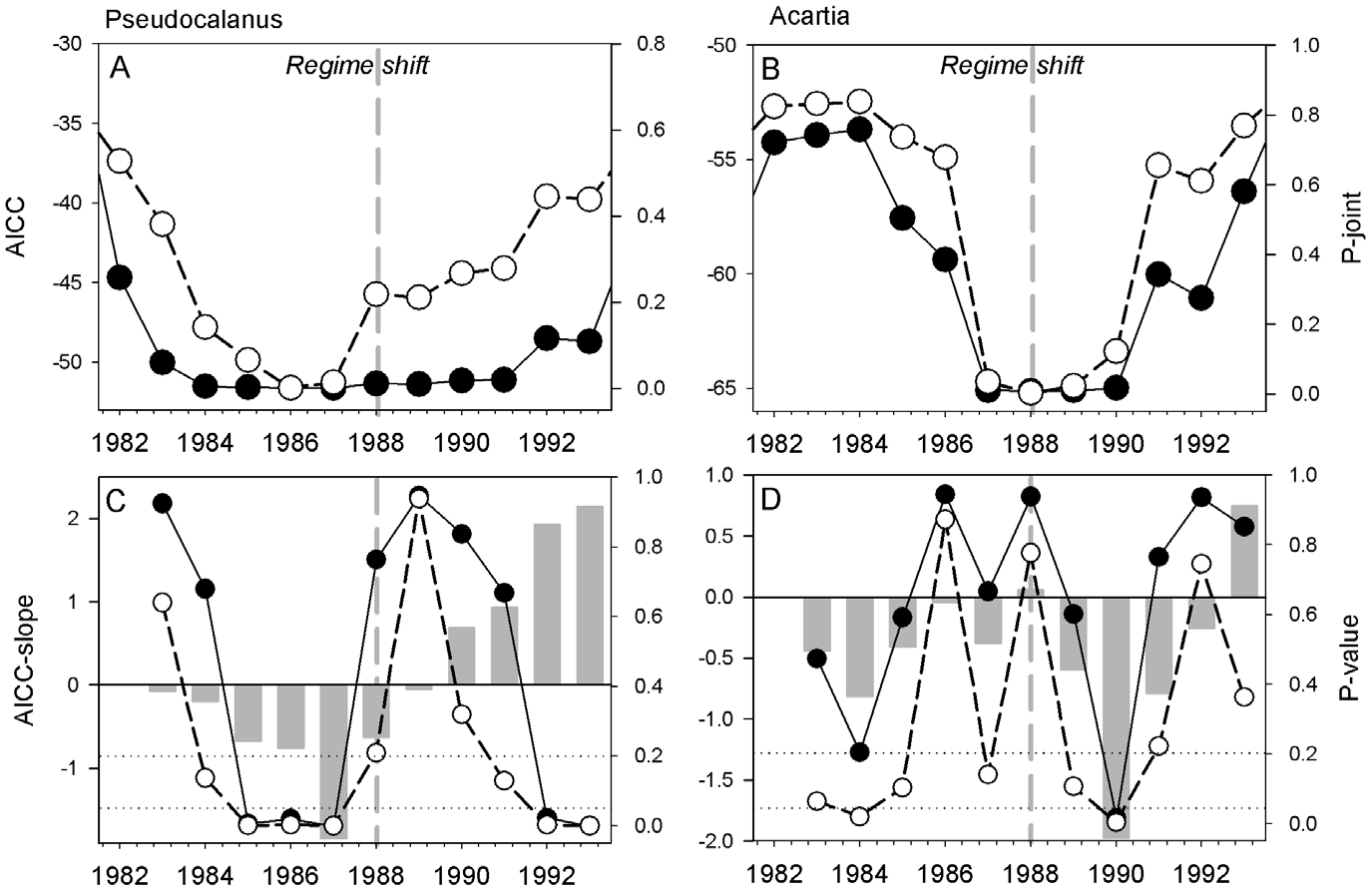
Shiftogram analysis of indicator time series. The shiftograms show the transition towards a local minimum in the AICC (black) and p-joint (white) for *Pseudocalanus acuspes* (A) and *Acartia* spp. (B). In panel (C, D), an alertogram demonstrates the AICC slopes (vertical bars), the estimated probabilities (p-values) of false warnings (white) and false no warnings (i.e., the beta error, black) over a 5-year period before and after the regime shift. The two dotted horizontal lines represent the significance level (p = 0.05) and the upper tolerance limits with regard to the probabilities of false detections (i.e., false alarm limit).

### Shiftograms

The third approach was not primarily designed for early detection either, but rather for the identification and detection of regime shift. It is based on the evaluation of statistical time series models including structural breaks and combines several statistical indicators into a so-called “shiftogram” [36]. The shiftogram approach is an iterative procedure combining econometric time series analysis and quantile methods displaying the gradual or rapid transition towards a local minimum (i.e., structural break-point) by making use of time series features and quality-of-fit criteria, such as the corrected Akaike’s information criterion (AICC) and a joint significance test (p-joint) of all parameters related to a particular type of structural break. These quality-of-fit criteria may be regarded as indicators of an imminent shift, illustrated by a potential decrease in AICC and p-joint statistics prior to a shift. Moreover, we used the AICC and p-joint test into an “alertogram” that primarily uses the negative slope of the AICC or p-joint values prior to a potential structural break by fitting a linear regression and performing assessments of false positives (type I error; probabilities of false warning) and false negatives (type II error; probabilities of false no-warnings) based on slope F tests. As neither the trend analysis nor the shiftogram approaches are developed as strictly early-detection methods, but for shift identification, we also refitted the GAMs and shiftograms on shortened time series until the major regime shift in 1988, in order to test whether they can be used to give early-warning, or whether they simply detect the shift once it is underway or even after it has occurred.

### Method Assessment

The full set of early-warning methods were assessed in terms of (i) the potential for detecting early-warnings signals in the selected indicator time series, (ii) how far in advance early-warning signals could potentially be detected, (iii) associated methodological assumptions and drawbacks influencing early-detection and (iv) applicability to real ecosystem management in terms of data requirements. While the trend analysis and shiftogram approach may quantitatively evaluate the first two criteria (i.e., by performing statistical tests), no predefined reference levels exist to objectively assess the performance of our ecological indicators of critical slowing down, nor the possibility to theoretically crash test the methods against a simulated (modeled) spatio-temporal data set [12]–[16]. In order to minimize the extent to which subjectivity and expert judgement influence the interpretation and assessment of our results, we argued that a potential signal may be alerted when an indicator value exceeds or falls below the upper and lower 95% confidence interval of its historical (cumulative) distribution prior to a regime shift. However, note that the above exercise is not suited for a direct comparison between methods *per se* but to assess how under the constraints of real ecological time series similar to ours, different approaches may or may not work, and which assumptions and drawbacks may pose the greatest challenges in terms of early-warning detection and applicability to management.

## Results and Discussion

### Indicators of Critical Slowing Down

Temporal variance of *Pseudocalanus acuspes* (measured as standard deviation, SD) strongly increased two years before the regime shift in 1988 (Figure 2A), but still remained below the upper confidence interval of its historical distribution. In the case of *Acartia* spp. the temporal variance also increased prior to the regime shift, but exceeded the upper confidence interval first after the shift had occurred (Figure 2B). Overall the strength of the increase in SDs depended on the number of years used for the sliding window, being strongest when using a 10 year window (Figure S2). On the contrary, temporal autocorrelation analysis demonstrated a marked and significant decline in AR(1) parameters (below the lower confidence level) preceding or coinciding with the regime shift (Figure 2C–D); regardless of the number of years used for the sliding window (Figure S2). Since early-warning detection depends on the choice of metric, the use of sliding windows, and constraints in the length of the time series, the potential of temporal indicators of critical slowing down for early-warning may be limited.

In contrast to the temporal analysis, spatial approaches for detecting patterns in either variance or correlation yielded rather similar results. The spatial variance in *Pseudocalanus acuspes* displayed strong inter-annual fluctuations and a significant increase in the coefficient of variation (CV) one year ahead of the regime shift (Figure 3C), while *Acartia* spp. showed decreasing CV below the lower confidence interval only after the shift (Figure 3B). The reason for opposite patterns in spatial CVs may originate from the pronounced differences in abundance trends, i.e., illustrating a decrease in *Pseudocalanus acuspes* (Figure 1B) and increase in *Acartia* spp. (Figure 1C), where decreasing abundances may simply increase spatial CVs and vice versa. Furthermore, our results show a continuous increase and decrease in CVs throughout the period, thus partly inconsistent to the theoretical expectation of critical slowing down, which predicts a decrease in variance after a shift as the system reaches its new equilibrium. Whether simply driven by the long-term abundance trends or caused by dynamics not yet having reached (stable) equilibrium, the discrepancies between theory and practical application deserve further attention.

The spatial (Moran’s I) correlation showed p-values exceeding their upper confidence intervals one year in advance of the regime shift for both species (Figure 3C–D). This may indicate a strong fragmentation of the zooplankton distribution well before the population sizes changed prior to the regime shift (e.g., even 6–7 years before the shift for *Pseudocalanus acuspes*; Figure S3A). In the case of *Pseudocalanus acuspes* this can be explained by the distribution of adults in deep water layer confined by oxygen conditions from below and salinity conditions from above [28], [29]. Reduction of oxygen and salinity levels due to a lack of inflows from the North Sea since the early 1980s, a major cause of the Baltic ecosystem regime shift [20], reduced the spatial extent of suitable reproductive habitat for the copepod. Hence, habitat fragmentation may have caused parts of the population to become spatially isolated from each other which may have impaired reproductive capabilities and resulted in the population decline [26]. Nevertheless, it has to be noted that our knowledge on the spatial dynamics of *Pseudocalanus acuspes* and *Acartia* spp. is still limited. We are therefore unable to provide a solid interpretation of the observed distribution patterns. Hence, the elevated heterogeneity in the distribution pattern, i.e., the consecutive peaks in p-values during the late-1980s (Figure S3A), may simply represent the natural spatial variability in the dynamics of Baltic Sea copepods and thus render the derived early-warning signals as potential false alarms.

**Table 1:** Assessment of early-detection methods in terms of (i) the potential for detecting early-warnings signals in the selected time series, (ii) how far in advance early-warning signals could potentially be detected (i.e., in number of years before the regime shift), (iii) major associated methodological assumptions and drawbacks influencing early-warning detection and (iv) applicability to real ecosystem monitoring and management in terms of data needs.

Method		i	ii	iii	iv
**1. Indicators of critical slowing down**					
A. Temporal variance		Medium	1–2	Size of window, Length of time series	Long-term data, slow variables
B. Temporal AR(1)		Low	0–1	Size of window, Length of time series	Long-term data, slow variables
C. Spatial variance		Low	0–1	Uneven sampling (No./distribution)	Spatial data, consistent design
D. Spatial r (Moran’s I)		Medium	1	Uneven sampling (No./distribution)	Spatial data, consistent design
**2. Trend analysis**					
A. Temporal GAM (I)		Low	(1)	Degrees of smoothers, “retrospective” (*f* ’’)	Recent trend, pressures/drivers
B. Temporal GAM (II)		Low	(1)	“retrospective” analysis, *f* ’’ calculation (t+1)	Recent trend, pressures/drivers
**3. Shiftograms**					
A. Shiftogram (AICC)		High	2–4	Gradual/rapid decrease, global vs local minima	Time series, contrasts/variability
B. Shiftogram (p-joint)		High	2–4	Broad local minima, timing of shift	Time series, contrasts/variability
C. Alertogram		Medium	1	Slope, power, significance of shifts in AICC	Time series, contrasts/variability

Contrary to temporal indicators of slowing down, spatial approaches for detecting changes in correlation and variance patterns [13], [14], [17] are not primarily constrained by methodological assumptions associated with a particular method, but are influenced by the quality and consistency of monitoring programs in space and time. However, our spatial analysis of critical slowing down seems robust to the random resampling of monitoring stations, e.g., spatial correlation between stations in each year showed consistent dynamics based on repeated random draws of 6, 8 and 10 stations, respectively (Figure S3), indicating that potential bias from uneven sampling between years may be of less importance or successfully accounted for by performing proper sensitivity analysis.

### Trend Analysis

The trend analysis [34] indicated potential early-warning signals given by a significant decreasing and increasing trend for *Pseudocalanus acuspes* and *Acartia* spp. prior to the regime shift (Figure 4). When refitted to the time-period preceding the regime shift (1960–1987), significant change points were detected between 1986 and 1987 (Figure S4), while when excluding year 1987 no change was detected before the regime shift. In the complimentary trend analysis [35], applying a routine for selecting the optimal numbers of regression splines, a significant increase in the rate of decline was indicated between 1985 and 1987 (Table S1), as illustrated by a negative *f′* (slope) and a positive *f′′* (acceleration). As in the previous example, no significant trend or change point was detected when excluding also 1987.

The trend analysis seems to be highly dependent on the length of time series and the numbers of regression splines used during fitting of the generalized additive models (GAMs; Figure 4). These assumptions influence the degree to which potential signals may resemble true early-warning signals, hence limiting the robustness of advice originating from such analysis. As an example of potential methodological bias, the choice of the numbers of regression splines results in differences in the number of change points detected, as well as in the timing of these changes (Figure 4). Even when statistically optimizing the number of splines [35], the length of the time series may influence the number and timing of significant change points being detected. However, it should be noted that trend analysis approaches were not primarily designed for early detection of regime shifts, but as an highly effective tool for detecting recent trends and change points in ecological time series [34], [35].

### Shiftograms

The shiftogram generally resulted in an early detection of regime changes well in advance of the regime shift. For *Pseudocalanus acuspes* both the Akaike’s information criterion (AICC) and the p-joint significance test showed a gradual transition towards a local minimum (i.e., structural break point) 3–4 years before the regime shift (Figure 5A), while for *Acartia* spp. an abrupt transition occurred only 2 years in advance (Figure 5B). This was the case regardless of fitting to the entire time series or to the time-period preceding the regime shift. In both cases, the p-joint statistics decreased before the AICC and remained on low values over a longer time-period. In addition, performing tests on false positive and negative warnings using a slope F test based on the AICC (alertogram), shows that two years before the local minimum in 1987 was reached, the negative decrease towards a break becomes highly significant (p<<0.05) in terms of the estimated slopes (Figure 5C). Thus, the year 1985 sharply marks the beginning of a gradual shift in *Pseudocalanus acuspes* with a clear alert signal. In addition, both type I and II errors exceed their upper significance limits before 1985 and after 1987, indicating that false positive and negative warnings occur outside the 1985–1987 period (Figure 5E). In contrast, the *Acartia* spp. time series displayed a significant alert signal first in 1990 (Figure 5D), despite a pronounced decrease in AICC and p-joint prior to the regime shift (Figure 5B).

The shiftogram approach appeared to be promising in detecting structural breakpoints well in advance before the regime shift. However, the way these metrics approach a local minimum are influenced by the type of transition at hand in the time series. Sudden (pulse) transitions or more gradual changes strongly influence the shape (e.g., steepness and size) of the local minimum and hence the degree to which these transitions can be detected sufficiently in advance; a difference illustrated by the abrupt decrease in *Acartia* spp. (Figure 5B) and the more gradual decline in *Pseudocalanus acuspes* (Figure 5A). In addition, a local minimum of considerable width and little steepness may saturate the value and hence the reliability of the derived early-warning signal. However, the use of alertograms may compliment the shiftogram approach by adding valuable information for decision support, such as the strength of the negative slope of the AICC, as well as the significance (false positive signals) and the power (false negative signals) of this slope. Because the alertogram displays the false warning probabilities along with the false no-warning probabilities it aids in evaluating the urgency of potential management actions.

### Conclusions

The versatility of methods for early detection of regime shifts in ecological time series provide an important toolbox for scientists and ecosystem managers. As learned from our example, no ‘one-size-fits all’ solution to deriving and interpreting spatio-temporal patterns announcing critical transitions exists [3]. Given by the type of transitions (i.e., sudden (pulse) transitions or more gradual changes) in the chosen ecological time series, some indicators and methods may result in clear early-warning signals, as demonstrated by the shiftogram and alertogram approaches, while other methods may have limited utility in informing ecosystem-based management, as they show no or weak (i.e., too late for a management measure to implement) early-warning potential (Table 1).

Hence, we suggest that a multiple method approach may provide a sound scientific basis for detecting and evaluating early-warning signals and thus provide timely advice for immediate management actions in the face of future ecosystem changes [37]. Such a multiple method approach should be based on (i) the availability and quality of monitoring data; (ii) a thorough sensitivity analysis of key methodological assumptions and potential sources of bias of a given methodology; and (iii) a scientifically sound interpretation of results based on the best available knowledge concerning the ecological variable in question. Eventually, early-warning systems, including suitable indicators and related methods, for detecting and preventing unwanted catastrophic changes must be tailored to the local ecosystem characteristics.

## Supporting Information

Figure S1 **Map of the Baltic Sea and its location within Northern Europe.** The central part of the Baltic Sea encompasses three deep (<70 m) basins important for marine biota, the Bornholm Basin (BB), the Gdansk Deep (GD) and the Gotland Basin (GB); largely corresponding to the International Council for the Exploration of the Sea (ICES) official sub-divisions 25, 25 and 28, respectively (thin lines). Furthermore, these basins are part of a long-term spatially and temporally disaggregated zooplankton monitoring program in the Baltic Sea.
(JPG)

Figure S2 **Temporal variance of** ***Pseudocalanus acuspes*** **(circles) and** ***Acartia*** **spp. (triangles) estimated by standard deviations (SD) and the first-order autocorrelation coefficient (AR(1)) of detrended time-series for a sliding window of 10 (A, D), 15 (B, E) and 20 (C, F) years.** Vertical dashed bars mark the timing of the Central Baltic Sea regime shift in the late 1980s.
(TIF)

Figure S3 **The degree of spatial correlation for** ***Pseudocalanus acuspes*** **(A) and** ***Acartia*** **spp. (B) estimated as the mean significance (p-value) of spatial correlation coefficients derived from a Moran’s I test across 6 (black), 8 (grey) and 10 (black) randomly assigned stations (after 1000 resamples).** Vertical dashed bars mark the timing of the Central Baltic Sea regime shift in the late 1980s.
(TIF)

Figure S4 **Smoothed indicator time-series of** ***Pseudocalanus acuspes*** **and** ***Acartia*** **spp. with GAM df = 10 (A, B) and df = 20 (C, D) from 1960–1987.** Bootstrapped confidence intervals are shown by grey lines. Acceleration in the rate of change (slope) in each year are shown by statistically significant second derivatives (*f′′*’), where black and white dots represent major downward- and upward trends, respectively.
(PPTX)

Table S1 **Test results for recent trends and changes in trends over 3-year periods before the regime shift in 1988 using intersection–union tests.** P-values from a χ^2^ goodness-of-fit test indicate whether the GAM fits satisfactory to the entire time-series. Significant negative (−) or positive (+) time trends in the rate of change (*f*′), as well acceleration (+) or deceleration (−) of the current trend (*f*′′) are shown. Hence, an increase in a rate of decline is indicated by negative *f*′ and a positive *f*′′.
(DOC)
