# Supplementary figures and images for: Early Detection of Ecosystem Regime Shifts: A Multiple Method Evaluation for Management Application
Source: PLoS One. 2012 Jul 10;7(7):e38410. doi: 10.1371/journal.pone.0038410 (PMC3393716; doi:10.1371/journal.pone.0038410)

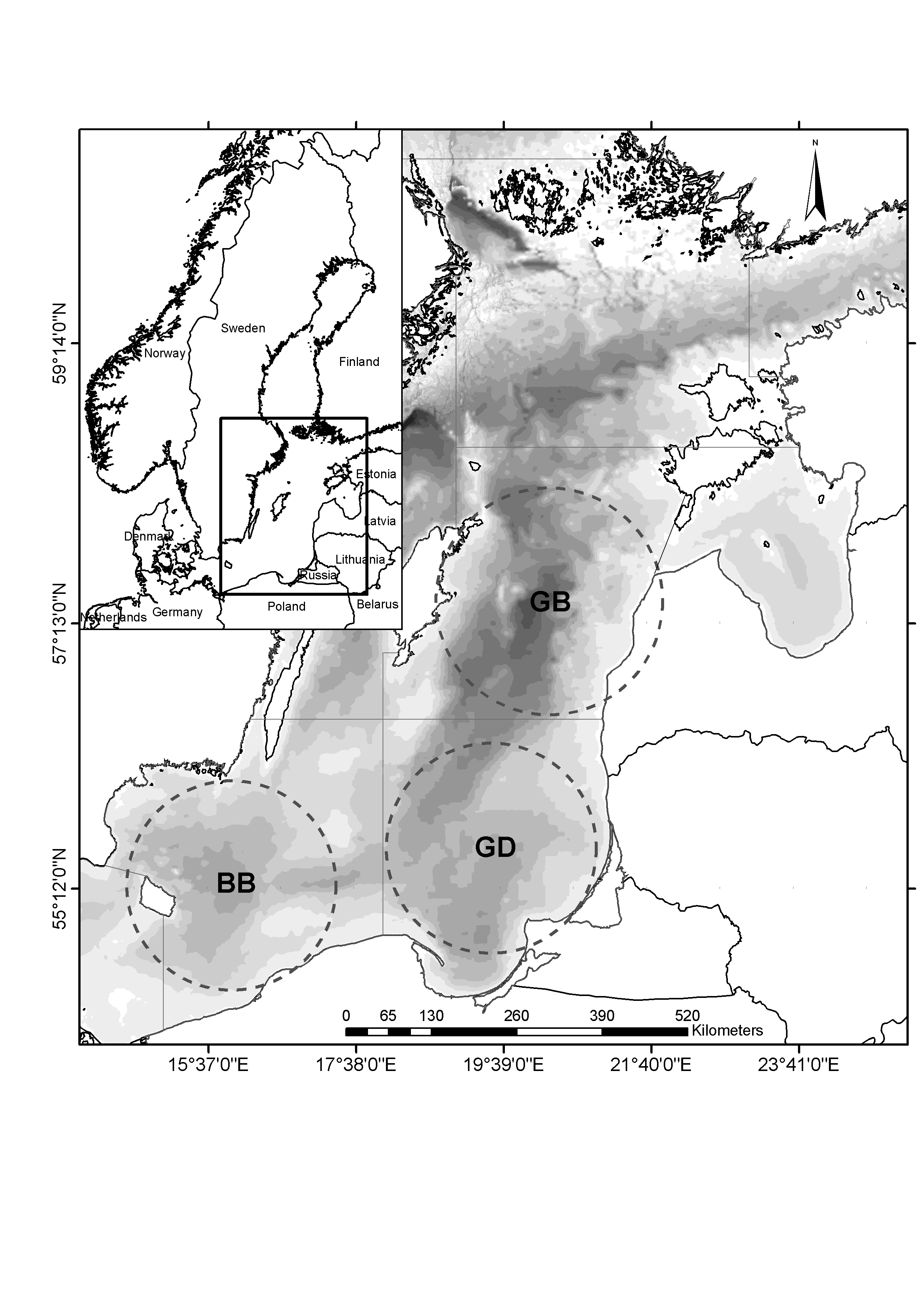

Supplement: Figure S1 — Map of the Baltic Sea and its location within Northern Europe. The central part of the Baltic Sea encompasses three deep (<70 m) basins important for marine biota, the Bornholm Basin (BB), the Gdansk Deep (GD) and the Gotland Basin (GB); largely corresponding to the International Council for the Exploration of the Sea (ICES) official sub-divisions 25, 25 and 28, respectively (thin lines). Furthermore, these basins are part of a long-term spatially and temporally disaggregated zooplankton monitoring program in the Baltic Sea. (JPG) [file pone.0038410.s001.jpg]

## Slide 1
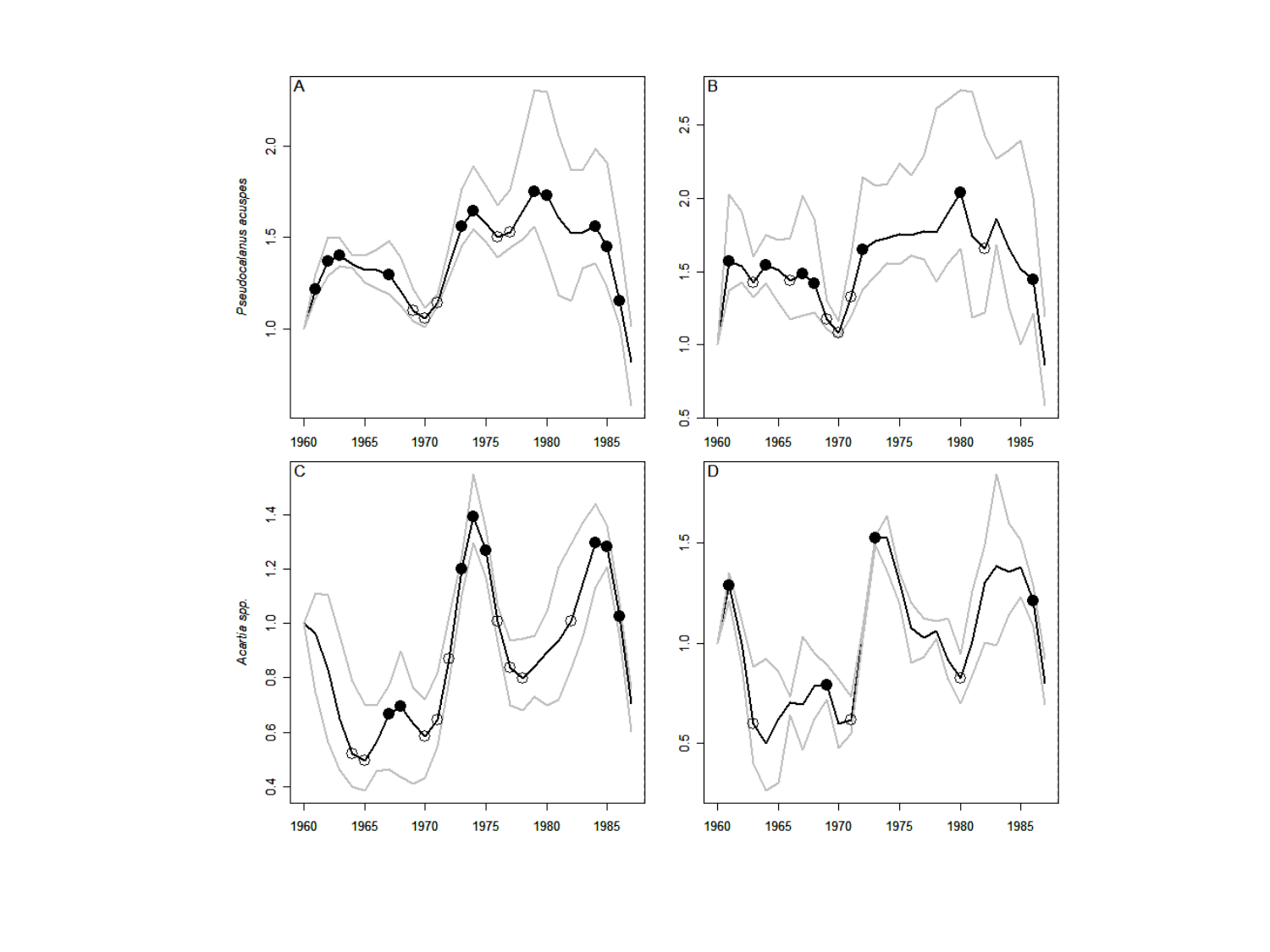

Supplement: Figure S4 — Smoothed indicator time-series of Pseudocalanus acuspes and Acartia spp. with GAM df = 10 (A, B) and df = 20 (C, D) from 1960–1987. Bootstrapped confidence intervals are shown by grey lines. Acceleration in the rate of change (slope) in each year are shown by statistically significant second derivatives (f′′’), where black and white dots represent major downward- and upward trends, respectively. (PPTX) [file pone.0038410.s004.pptx]
