# Supplementary material for: Early Detection of Ecosystem Regime Shifts: A Multiple Method Evaluation for Management Application
Source: PLoS One. 2012 Jul 10;7(7):e38410. doi: 10.1371/journal.pone.0038410 (PMC3393716; doi:10.1371/journal.pone.0038410)
Supplement: Table S1 — Test results for recent trends and changes in trends over 3-year periods before the regime shift in 1988 using intersection–union tests. P-values from a χ2 goodness-of-fit test indicate whether the GAM fits satisfactory to the entire time-series. Significant negative (−) or positive (+) time trends in the rate of change (f′), as well acceleration (+) or deceleration (−) of the current trend (f′′) are shown. Hence, an increase in a rate of decline is indicated by negative f′ and a positive f′′. (DOC) [file pone.0038410.s005.doc]

**Table S1** Test results for recent trends and changes in trends over 3-year periods before the regime shift in 1988 using intersection–union test (IU, 11). P-values from a χ^2^ goodness-of-fit test indicate whether the GAM fits satisfactory to the entire time-series. Significant negative (-) or positive (+) time trends in the rate of change (*f*´), as well acceleration (+) or deceleration (-) of the current trend (*f*´´) are shown. Hence, an increase in a rate of decline is indicated by negative *f´* and a positive *f´*´.

Start year End year χ^2^ (p-value) Trend (*f´*) Change (*f´´*)

*Pseudocalanus acuspes*

1985 1987 <0.001 - +

1984 1986 <0.001 0 0

*Acartia* spp.

1985 1987 <0.001 - +

1984 1986 <0.001 0 0
